# Transcriptome analysis of extended-spectrum β-lactamase-producing *Escherichia coli* and methicillin-resistant *Staphylococcus aureus* exposed to cefotaxime

**DOI:** 10.1038/s41598-018-34191-3

**Published:** 2018-10-30

**Authors:** P. R. Brochmann, A. Hesketh, B. Jana, G. H. Brodersen, L. Guardabassi

**Affiliations:** 1 University of Copenhagen, Faculty of Health and Medical Sciences, Department of Veterinary and Animal Sciences, Frederiksberg, Denmark; 2 University of Cambridge, Department of Biochemistry and Cambridge Systems Biology Centre, Cambridge, United Kingdom; 3 University of Brighton, School of Pharmacy and Biomolecular Sciences, Brighton, United Kingdom

## Abstract

Previous studies on bacterial response to antibiotics mainly focused on susceptible strains. Here we characterized the transcriptional responses of distinct cephalosporin-resistant bacteria of public health relevance to cefotaxime (CTX), a cephalosporin widely used in clinical practice. Adaptation to therapeutic concentrations of CTX (30 µg/ml) was investigated by RNA sequencing in mid-exponential phase cultures of a methicillin-resistant *Staphylococcus aureus* (MRSA) and two genetically diverse *E. coli* producing CTX-M-15 or CMY-2 β-lactamase following genome sequencing and annotation for each strain. MRSA showed the most notable adaptive changes in the transcriptome after exposure to CTX, mainly associated with cell envelope functions. This reprogramming coincided with a transient reduction in cell growth, which also occurred in the CMY-2-producing *E. coli* but not in the CTX-M-15-producing strain. Re-establishment of growth in the CMY-2 producer proceeded without any notable adaptive transcriptional response, while limited reprogramming of gene transcription was observed in the CTX-M-15 producer. Our data show that the transcriptional response of CTX-resistant bacteria to CTX depends on the bacterial species, level of resistance and resistance determinant involved. Gene products induced in the presence of CTX may play an essential role for bacterial survival during therapy and merit further investigation as possible targets for potentiating CTX.

## Introduction

Oxyimino-cephalosporins such as cefotaxime (CTX) are classified by the World Health Organization (WHO) amongst the “highest priority critically important antimicrobials” for human medicine^1^. They are safe and broad-spectrum β-lactam antibiotics that play a pivotal role in the management of infections caused by both Gram-negative and Gram-positive pathogens. Resistance to these agents is mediated by a variety of mechanisms depending on the specific microorganism involved. The most common mechanism in *Escherichia coli* and other Gram-negative pathogens is enzymatic drug degradation via production of extended-spectrum β-lactamases (ESBLs) belonging to different functional and structural classes^2^. ESBLs are mainly disseminated by horizontal transfer of plasmids across diverse *E. coli* lineages^3^, but also via dissemination of certain high-risk clones such as sequence type (ST) 131, which has significantly contributed to the global spread of CTX-M-15 and other ESBL types^4^. Among Gram-positive pathogens, *Staphylococcus aureus* and other staphylococci have developed a completely different mechanism of resistance that makes them clinically resistant to all conventional β-lactams, including oxyimino-cephalosporins. In this case resistance is encoded by a large mobile chromosomal element (staphylococcal cassette chromosome *mec*, SCC*mec*) that carries the methicillin resistance gene *mecA* encoding a penicillin binding protein (PBP2a) with low affinity for β-lactams^5^. Methicillin-resistant *S. aureus* (MRSA) spread by dissemination of epidemiologically successful clones, including the livestock-associated clone belonging to ST398, which can be transmitted to humans by direct contact with livestock or environmental exposure^6^.

Previous studies have shown that bacterial responses to antibiotics include induction of both common and drug-specific changes in gene transcription^7–9^. Earlier studies on adaptation to antibiotics have focused on measuring gene expression profiles of susceptible bacteria exposed to antibiotics at sub-MIC^8^. Here we analyze the responses of ESBL-producing *E. coli* and MRSA strains to CTX. The aim was to elucidate the transcriptomic response of different cephalosporin-resistant bacteria of public health relevance after exposure to a CTX concentration mimicking antimicrobial therapy (30 µg/ml). Three well-characterized isolates were used as model strains: two *E. coli* strains producing CTX-M-15, a class A ESBL^2^ that is widespread among human clinical isolates^10,11^, and CMY-2, a class M ESBL^2^ that is common in both humans and animals^12–14^, respectively, and a MRSA strain belonging to the livestock-associated clone ST398. By using the selected strains, we gained insight into the stress responses of different types of bacteria exposed to the antibiotic they are resistant to. Bacterial cultures were exposed to CTX in mid-exponential phase and the transcriptome was profiled immediately before antibiotic exposure and 30 and 90 min after by RNA-sequencing. This approach included genome sequencing of all three strains in order to identify all genes putatively involved in CTX resistance and permit the subsequent analysis of the transcriptomes.

## Results

### Genetic potential for CTX resistance in the selected *E. coli* and *S. aureus* strains

Two ESBL-producing *E. coli* strains UR40 (ST131) and R7AC (ST297), and MRSA 55488 (ST398) were used as models for this study. *E. coli* UR40 harbors *bla*_CTX-M-15_ on an IncF plasmid and originated from a human patient with urinary tract infection^15^ while *E. coli* R7AC carries *bla*_CMY-2_ on a IncI1 plasmid (pR7AC) and was isolated from dog faeces^16^. MRSA 55488 harbors *mecA* on a SCC*mec* type Vc (5C2&5) and was isolated from a healthy pig farmer^17^. The three strains displayed the following MICs of CTX: >256 µg/ml (UR40), 16 µg/ml (R7AC) and 8 µg/ml (55488). The genome sequences for the two *E. coli* strains were determined and analysed alongside the sequence available for S. aureus 55844^17^ in order to obtain a complete picture of the genetic potential for CTX resistance in each strain. Table 1 lists the known genes conferring acquired clinical resistance to β-lactams. Additional genes putatively involved in β-lactam resistance, including regulatory genes, are listed in Supplementary File 1. The genome of *E. coli* R7AC contained two resistance genes coding for plasmid-mediated and chromosomal AmpC β-lactamase, *bla*_*CMY-*2_ (UG47_03805) and *ampC* (UG47_16890), respectively. The annotated sequences of the two genes are here the same. Thus, the gene names were assigned based on interpretations of expression results and knowledge from the literature about the genes.

**Table 1:** Known genes conferring acquired clinical resistance to β-lactams in the three strains under study.

Strain	Gene identifier	Gene name	Protein name	Link to β-lactam resistance	Genbank accession
*E. coli* R7AC	UG47_03805	*bla*_*CMY-2*_	CMY-2	β-lactamase	WP_001447737.1
	UG47_16890	*ampC*	AmpC	β-lactamase	WP_001447737.1
	UG47_05450	*bla*_*TEM-1*_	TEM-1	β-lactamase	WP_010331504.1
*E. coli* UR40	UG58_19655	*bla*_*CTX-M-15*_	CTX-M-15	β-lactamase	WP_000239590.1
	UG58_26285	*bla*_*TEM-1*_	TEM-1	β-lactamase	WP_010331504.1
	UG58_26550	*bla*_*OXA-1*_	OXA-1	β-lactamase	WP_032491311.1
	UG58_08585	*ampC*	AmpC	β-lactamase	WP_001336292.1
MRSA 55488	WG79_13605	*mecA*	PBP2a	Low-affinity penicillin-binding protein	WP_001801873.1
	WG79_09935	*blaZ*	BlaZ	β-lactamase	WP_002457930.1

### Effects of CTX on strain growth and viability

**Figure 1:**
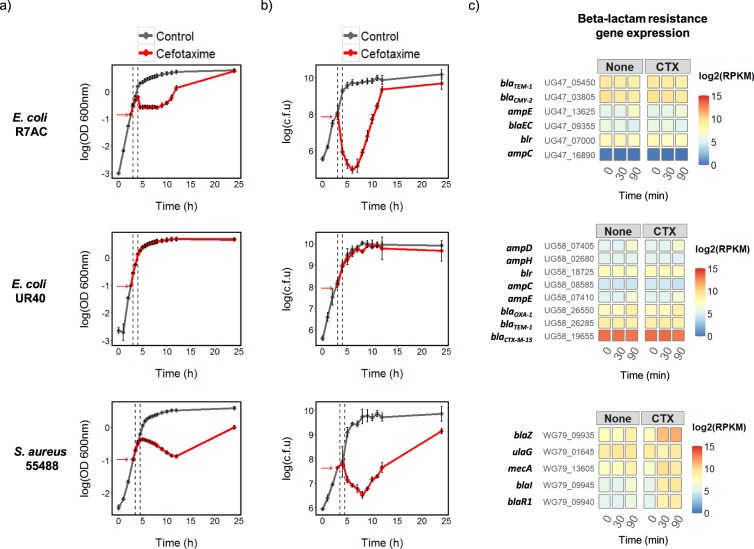
The effect of CTX (30 μg/ml) on (**a**) growth and (**b**) viability of CMY-2-producing *E. coli* R7AC, CTX-M-15-producing *E. coli* UR40 and MRSA 55488, and (**c**) its relationship to expression of genes putatively involved in CTX resistance (Supplementary File 1). For (**a**) and (**b**) addition of CTX to the cultures is arrowed (subsequently defined as 0 min), and the sampling points for the transcriptome analysis at 30 min and 90 min after antibiotic addition are indicated with dotted lines. The mean of log10 values from triplicate experiments (+/− standard deviation) are shown for (**a**) OD measurements at 600 nm, and (**b**) CFU from viability assays. The normalized log2(RPKM) mean abundance of transcripts (n = 3) putatively involved in CTX resistance in each strain for the 0, 30 and 90 min samples is shown in (**c**) (see Table 1 and Supplementary File 1). Constitutively high expression of UG58_19655 encoding CTX-M-15 is associated with the lack of sensitivity of strain UR40 towards CTX.

Triplicate cultures of each strain were exposed in mid-exponential growth phase to the same therapeutic concentration of CTX (30 μg/ml) and sampled to determine the effects on growth, viability and expression of the transcriptome as described in the Materials and Methods. Markedly different effects of CTX on growth and viability were observed based on the resistance level in each strain (Fig. 1). While CTX-M-15-producing *E. coli* UR40 (MIC > 256 µg/ml) was completely unaffected by CTX compared to the unexposed culture, CMY-2-producing *E. coli* R7AC (MIC = 16 µg/ml) and the MRSA strain (MIC = 8 µg/ml) showed a temporary decrease in both culture optical density and viable counts in the 1 to 6 hour period following CTX addition (Fig. 1a,b). The CMY-2-producing *E. coli* eventually recovered to approximately the levels seen in the unexposed controls, while the MRSA strain recovered to a lesser extent. The recovery process is likely to involve both adaptations in gene expression allowing improved survival and growth in the presence of CTX, and also stochastic heterogeneity in the bacterial cultures leading to the survival and eventual growth of sub-populations of more resistant cells. In the analysis of the changes in the transcriptome over the 90 min period immediately following CTX addition described below, we focus on the first of these two processes.

### Effects of CTX on expression of predicted β-lactam resistance genes

Analysis of the transcription of the genes directly involved in β-lactam resistance (Table 1) indicated very high and constitutive expression of *bla*_*CTX-M-15*_ (UG58_19655) in all samples of *E. coli* UR40 (Fig. 1c). This strain also exhibited significant constitutive expression of *bla*_*TEM-1*_ (UG58_26285) and *bla*_*OXA-1*_ (UG58_26550). Similarly, *bla*_*CMY-2*_ (UG47_03805) and *bla*_*TEM-1*_ (UG47_05450) showed constitutively high levels of transcription in *E. coli* R7AC. The chromosomal *ampC* was poorly expressed in UR40 and not transcribed at all in R7AC. Uniquely, CTX up-regulated transcription of genes directly specifying β-lactam resistance only in the MRSA strain (Fig. 1c). Transcription of *mecA* (WG79_13605) and *blaZ* (WG79_09935) was induced 3.8 and 11.3-fold (Supplementary File 2), respectively, 30 min after treatment. Transcription of the *blaZ* regulatory genes, *blaR1* (WG79_09940) and *blaI* (WG79_09945), were also immediately induced in response to CTX, exhibiting a similar profile of induction to that observed for *blaZ* (Fig. 1c).

### Effects of 30-min CTX exposure on the global transcriptional response

**Figure 2:**
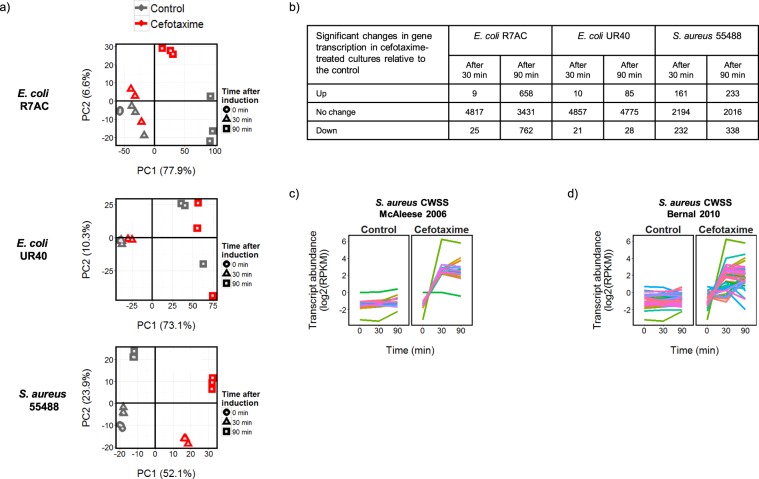
The transcriptome of MRSA 55488 responds more dynamically to CTX treatment than either of the *E. coli* strains. (**a**) PCA analysis of normalized transcript abundances (principal component 1 (PC1) v principal component 2 (PC2)) characterizes the global transcriptional changes taking place in response to both time and CTX treatment. The percentages given in brackets correspond to the proportion of the variability in the data explained by PC1 or PC2. (**b**) The numbers of transcripts significantly changed in abundance over the 30 and 90 min time periods following CTX exposure, relative to the unexposed control. Significance was determined using limma treat with an FDR ≤ 0.05 applied to a fold-change threshold of 1.5. Lists of differentially expressed transcripts are provided in Supplementary Files 2–4. The significantly differently expressed transcripts identified for 55488-ST398 include a marked and immediate up-regulation in transcription of the general cell wall stress stimulon (CWSS) previously identified by (**c**) McAleese *et al.*^18^ and (**d**) Bernal *et al.*^19^ (normalized log2(RPKM) mean abundance of transcripts are shown (n = 3) and plots have been mean centered). See Supplementary File 5 for details of the stress stimulon genes in 55488-ST398.

Principal components analysis (PCA) was used for unsupervised clustering of the RNA-sequencing data to produce an initial high-level comparison of the global transcriptional response to CTX occurring in each strain (Fig. 2a). By definition, the first principal component (PC1) explains the highest variability in the transcript abundance data being analyzed, while the second component (PC2) covers the second highest (etc). PCA can thus reveal global trends in the similarities and differences between the transcriptome data acquired for each sample. A clear separation in PC1 of the CTX-treated sample replicates from the untreated controls was observed for the MRSA strain, indicating a more defined transcriptional response to CTX in this Gram-positive strain than in the two *E. coli* strains (Fig. 2b). This observation was especially evident for the samples taken 30 min after CTX addition (Fig. 2a), and was consistent with CTX eliciting a more rapid transcriptional response in the *S. aureus* strain (Fig. 2b). The results from statistical testing supported this hypothesis (Supplementary Files 2–4). In MRSA 55488, 393 genes (~15% of the genome) showed significant [false discovery rate (FDR) ≤ 0.05 at a >1.5-fold change threshold] changes in transcript abundance in the CTX-exposed cultures relative to the control, while for *E. coli* UR40 and R7AC only 31 and 34 significant transcripts were identified, respectively (<1% of each genome).

### Effects of 90-min CTX exposure on the global transcriptional response

**Figure 3:**
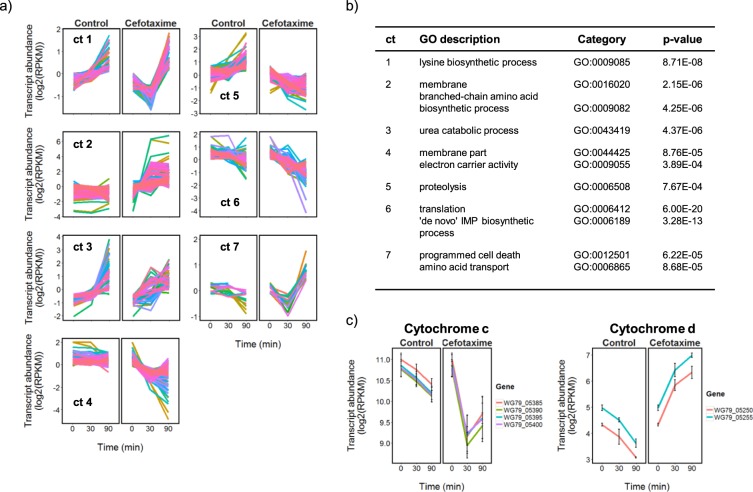
The transcriptional response of MRSA 55488 to CTX includes a down-regulation of genes required for growth, and a marked reorganization of cell envelope associated functions. The transcripts (n = 734) identified as being differentially expressed in response to CTX were clustered (**a**) and gene ontology (GO) enrichment analysis was applied to each cluster (ct) for functional classification (**b**). Cluster 4 contains a putative operon of genes encoding a cytochrome c oxidase, whose transcription contrasts with the predicted cytochrome d oxidase genes identified in cluster 2 (**c**). Normalized log2(RPKM) mean abundance of transcripts are shown (n = 3), and the plots in (**a**) but not (**c**) have been mean centered. Where shown, error bars correspond to +/− SD (n = 3). Cluster membership is listed in Supplementary File 6, together with the complete gene ontology analysis results.

Consideration of the samples taken 90 min after addition of CTX in the PCA analysis confirmed the establishment of a CTX-dependent transcriptional program in the MRSA strain. The MRSA adaptive response to CTX included a down-regulation of genes required for growth, and extensive changes in transcription associated with cell envelope functions. Exposure of the MRSA strain to CTX significantly altered the abundance of 734 unique transcripts in the 90 min period following addition of CTX, relative to the control cultures. Hierarchical clustering of the transcript abundance profiles grouped these into 7 different expression profiles, including three which show a marked down-regulation in expression in the CTX-exposed samples compared to the control (Fig. 3, Supplementary File 6; clusters 4, 5 and 6). The transcripts in cluster 6 encode proteins that are associated with translation (GO:0006412, p-value: 6.00E-20) and *de novo* inosine 5′-monophosphate (IMP) biosynthesis (GO:0006189, p-value: 3.28E-13). As both processes are required for cell growth, this result is consistent with the reduction in culture OD_600_ and viability observed after CTX addition (Fig. 1), indicating a programmed reduction in growth in response to the antibiotic challenge. The MRSA response also included a marked up-regulation of a large cluster of genes (cluster 2 in Fig. 3) showing functional enrichment for branched chain amino acid biosynthesis and membrane components. This cluster also contained the majority of the general cell wall stress stimulon genes^18,19^ and the β-lactamase locus WG79_09935-WG79_09945 (Supplementary File 6). Cluster 4 in Fig. 3 shows a significant coordinated down-regulation of genes encoding membrane components, which when considered alongside the results from cluster 2 indicated a complex reorganization of cell envelope functions in response to CTX. Interestingly, the data also indicate a shift away from using cytochrome c oxidase as the terminal electron acceptor in respiration following CTX treatment, towards using cytochrome d, suggesting a reduction in oxygen tension in the cultures arising from the CTX exposure (Fig. 3c). The small group of transcripts identified as being induced in abundance in response to CTX in cluster 7 shows functional enrichment for amino acid transport and programmed cell death, consistent with the induction of processes for recycling nutrients from cells terminally damaged by the antibiotic exposure.

**Figure 4:**
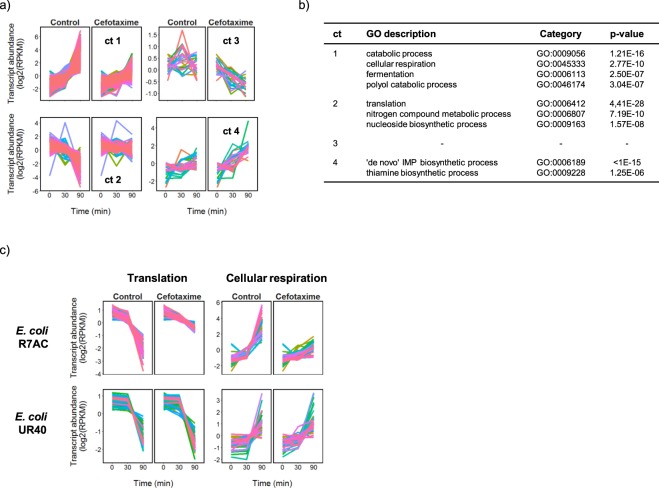
CTX-dependent changes in transcription in CMY-2-producing *E. coli* R7AC are associated with failure to enter transition phase in the 90 min period following CTX addition. The transcripts (n = 1454) identified as being differentially expressed between the control and CTX-exposed cultures were clustered (**a**) and gene ontology (GO) enrichment analysis was applied to each cluster (ct) for functional classification (**b**) Plots to contrast the transcript abundance measurements for the significant genes in R7AC-ST297 ct 2 that are present in the translation GO category, and R7AC-ST297 ct 1 in the cellular respiration category, with orthologues in strain UR40-ST131 are shown in (**c**). Normalized log2(RPKM) mean abundance of transcripts are shown (n = 3), and all the plots have been mean centered. Cluster membership is listed in Supplementary File 7, together with the complete gene ontology analysis results.

The majority of the CTX-induced changes in CMY-2-producing *E. coli* R7AC were associated with failure to enter stationary phase, and not with the initiation of an adaptive response. For this strain, the 90-min CTX-exposed samples are separated from all other samples by a combination of both PC1 and PC2, indicating a distinctive transcriptional profile. PC1 however primarily separated the 90-min control samples from the earlier 30-min control time points, demonstrating a significant time-dependent component to the changes in transcription occurring in these cultures. Differential expression analysis and transcript clustering were performed to characterize the difference between the behaviours of *E. coli* R7AC in the presence of CTX compared to the control condition (Fig. 4a, Supplementary File 7). Two large clusters of transcripts were identified which showed a marked increase (cluster 1) or decrease (cluster 2) in abundance in the control cultures that was absent (or much reduced) in the CTX-exposed cultures over the same 90 min period. The genes represented by cluster 2 are significantly enriched for functions associated with translation, nitrogen metabolism and nucleoside biosynthesis, while cluster 1 is enriched for catabolic processes and for respiration (Fig. 4b). The abundance of a relatively small number of transcripts was found to specifically change in response to CTX, as represented by clusters 3 and 4 in Fig. 4. Interestingly, cluster 4, which showed up-regulation in response to CTX, is significantly enriched for genes encoding enzymes for *de novo* IMP biosynthesis, and thiamine biosynthesis.

**Figure 5:**
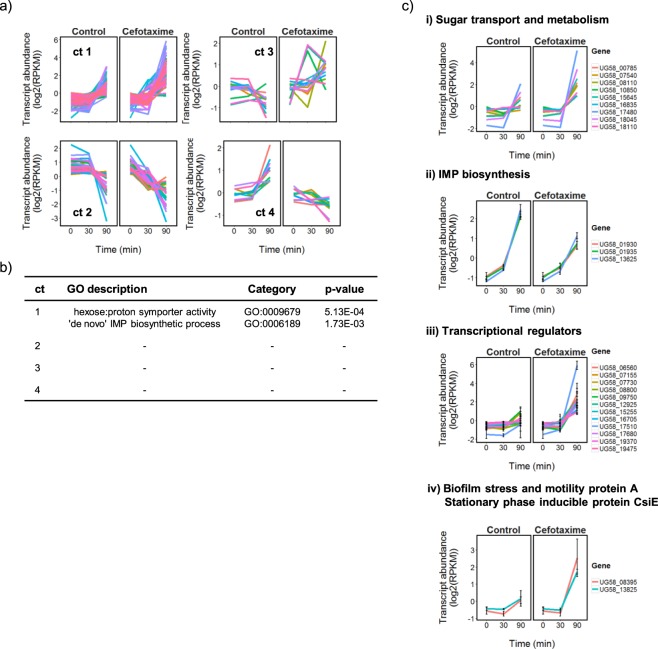
Limited effects on CTX-M-15-producing *E. coli* UR40 transcription in response to CTX. The transcripts (n = 113) identified as being differentially expressed in response to CTX were clustered (**a**) and GO enrichment analysis was applied to each cluster for functional classification (**b**). The mean of the log2(RPKM) normalized transcript abundances are shown (n = 3), and all plots have been mean centered. Transcription of genes highlighted by the GO analysis, or from selected genes of interest present in cluster 1, are shown (**c**). Error bars correspond to +/− SD (n = 3). Cluster membership is listed in Supplementary File 8, together with the complete gene ontology analysis results.

Only a small number of transcripts (113/4888, 2%) showed significant changes in abundance between the control and CTX-exposed cultures of CTX-M-15-producing *E. coli* UR40 strain after 90 min (Fig. 2 and Supplementary File 4). Clustering and functional analysis provided evidence for a CTX-dependent transcriptional response that involved expression of a number of genes with predicted functions related to sugar transport and metabolism, 12 genes encoding transcriptional regulators and two predicted stress inducible genes (Fig. 5c and Supplementary File 8). Three genes (UG58_01930, UG58_01935 and UG58_13625) encoding enzymes from the *de novo* IMP biosynthesis pathway were also notably down-regulated with respect to the control samples in the 90 min samples, although the remainder of the pathway was unaffected.

## Discussion

We examined the response of different types of cephalosporin-resistant bacteria of high clinical relevance, namely ESBL-producing *E. coli* and MRSA, after exposure to a high concentration of CTX (30 μg/ml) that is achieved in serum after standard dosage. The inhibitory effects of CTX on growth of the CMY-2-producing *E. coli* and MRSA strains were expected since the CTX concentration used exceeded the MICs of CTX in the two strains (16 and 8 µg/ml, respectively). The higher susceptibility of these two strains explains the significant changes in abundance of transcripts between control and CTX-exposed cultures (1454/4851 (30%) and 964/2587 (37%) respectively, Fig. 2b and Supplementary Files 2, 3). These transcripts belonged to various functional categories, which included catabolic, cellular respiration, fermentation, polyol catabolic, translation, nitrogen compound, metabolic, nucleoside biosynthetic, *de novo* IMP biosynthetic and thiamine biosynthetic processes for CMY-2-producing *E. coli* (Fig. 4 and Supplementary File 7); and lysine biosynthetic, membrane, branched-chain amino acid biosynthetic, urea catabolic, membrane part, electron carrier activity, proteolysis, translation, *de novo* IMP biosynthetic, programmed cell death, amino acid transport processes for MRSA (Fig. 3 and Supplementary File 6). Altogether, these data show a clear relationship between the CTX resistance phenotype and transcriptional response of resistant bacteria to CTX. However, the response of each strain was unique, suggesting that factors other than the MIC are responsible for modulating the transcriptional response, such as the nature of the bacterial cell wall (Gram-positive vs Gram-negative), the type of resistance mechanism (acquisition of a low-affinity target vs enzymatic degradation) and the activity of the specific β-lactamase involved (BlaZ vs CMY-2 vs CTX-M-15).

The MRSA transcriptome responded more rapidly and dynamically to CTX than the two *E. coli* strains. The significant changes in MRSA gene expression included all of the cell wall stress stimulon genes initially identified by McAleese *et al*.^18^, and 44/58 of those in the extended stimulon subsequently proposed by Bernal *et al*.^19^ (Fig. 2c,d). The stimulon corresponds to a group of genes induced in *S. aureus* following exposure to a variety of cell wall active antibiotics, including vancomycin, oxacillin, bacitracin and daptomycin. Activation of the cell wall stress stimulon by CTX is consistent with a rapid CTX-dependent alteration in the transcriptional program of MRSA that is apparently absent in the *E. coli* strains. CTX did not significantly affect transcription of the ‘accessory gene regulator’ operon *agrBDCA* (WG79_09750 - WG79_09735), but a general decrease in transcript abundance was observable following CTX addition (Supplementary Fig. 1). The *agr* gene regulatory system is important for pathogenicity and virulence in MRSA^20^ and a recent study found evidence for a trade-off between virulence regulation by *agr* and the regulation of β-lactam antibiotic resistance whereby transcription of the *agr* operon was repressed in the presence of nafcillin^21^. This was attributed to the presence of a potential MecI/BlaI operator binding site overlapping the *agr* operator sites in the *agrB* promoter and it is interesting to note that the strain used in this study shares the same sequence arrangement (Supplementary Fig. 1).

CTX treatment induced only limited changes in the transcriptome of *E. coli* UR40 (Fig. 2). This is consistent with high-level constitutive expression of CTX-M-15 β-lactamase in this strain which apparently helps to provide complete protection against CTX at the phenotypic level (Fig. 1). Constitutive expression of *bla*_*TEM-1*_ (UG58_26285) and *bla*_*OXA-1*_ (UG58_26550), but not *ampC* (UG58_08585), likely also contributes to this protection (Fig. 1). Evidence for CTX-induced adaptations in internal cell physiology was however obtained, with changes in sugar transport and metabolism, IMP biosynthesis, and gene regulation identified as part of the response to CTX (Fig. 5 and Supplementary File 8). We hypothesize that these adaptations may be important for surviving the effects of the antibiotic even in strains expressing high-levels of CTX resistance (MIC > 256 µg/ml).

Constitutive expression of the β-lactamase resistance genes *bla*_*CMY-2*_ (UG47 03805) and *bla*_*TEM-1*_ (UG47_05450) was insufficient to provide the R7AC strain with the level of protection from CTX that was observed in the UR40 strain (Fig. 1). Specific reprogramming of gene expression in response to CTX in strain R7AC was also limited. The majority of transcripts changing significantly in abundance between the control and CTX-treated cultures correspond to changes expected on entry into transition phase (eg. down-regulation of transcription of genes encoding the translational apparatus) taking place in the control cultures over the 90 min period of the experiment, but not occurring in the CTX-treated cultures. This suggests an arrest of growth following CTX treatment that is not dependent on the stringent control of translation. Significant changes in transcription of genes required for IMP biosynthesis were however again observed and this is discussed in more detail below. *E. coli*, like *S. aureus*, has regulatory systems to respond to cell envelope and other stresses. Miller *et al*. reported an SOS cell division inhibition response induced via the *dpiAB* two-component regulatory system by exposure of a susceptible *E. coli* strain UT481 to sub-lethal concentrations of ampicillin^22^. Jozefczuk *et al*. also characterized a general stress response in *E. coli* which includes common changes in expression of the transcriptome in response to cold shock, heat shock, oxidative stress, lactose diauxie and entry into stationary phase^23^. CTX treatment of the *E. coli* strains used in this study interestingly did not reproduce either of these responses. This is consistent with a previous study analyzing CTX-induced stress in a CTX-M-1-producing *E. coli* MG1655 genetic background^24^.

The transcription of genes involved in *de novo* IMP biosynthesis showed notable strain-to-strain variability both in the changes in expression with time and in response to CTX treatment, despite growth in the same culture medium (Supplementary Fig. 2). This pathway was also reportedly significantly up-regulated following treatment of *E. coli* MG1655 strains expressing CTX-M-1 β-lactamase resistance with sub-inhibitory concentrations of CTX^24^. Purine nucleotide biosynthesis is required for cell growth and proliferation, supplying precursors for the synthesis of DNA and RNA, and for ATP and GTP biosynthesis for cellular energy metabolism. In *E. coli* UR40 the genes involved in IMP biosynthesis were induced equally in both the CTX-treated and control cultures as the cultures aged, consistent with a gradual depletion of purines from the medium and an increased demand for *de novo* synthesis. In contrast, the significant increase in transcription of genes required for IMP biosynthesis 90 min after CTX exposure in *E. coli* R7AC, and in the previous report of CTX-M-1-producing *E. coli* MG1655 treated with CTX^24^, may reflect the increased need for nucleotide precursors for overcoming CTX stress and resuming growth in these strains. The reason for the increased demand for purine nucleotides following CTX treatment is unknown but it may at least in part be attributable to perturbations in energy use or increases in DNA repair mechanisms. Exposure of *E. coli to* bactericidal antibiotics such as CTX is known to increase rates of respiration^25^ and induce reactive oxygen species (ROS) leading to DNA damage^26,27^. A recent metabolomics study of *E. coli* exposed to a lethal concentration of bactericidal antibiotics showed a decrease in the abundance of nucleotide precursors within cells which was attributed to an increase in nucleotide turnover arising from DNA damage^27,28^. β-lactam antibiotics have also been shown to induce a toxic malfunctioning of peptidoglycan biosynthesis in *E. coli*, causing a futile cycle of cell wall synthesis and degradation and thereby depleting cellular energy resources and promoting cell death^29^. In contrast to *E. coli*, CTX-dependent induction of IMP biosynthetic genes was not observed in *S. aureus* 55844 in the present study. Indeed, genes required for the IMP pathway were significantly down-regulated both 30 and 90 min after exposure to CTX in this strain. While this may reflect an increase in purine nucleotide availability due to cell lysis, or a reduced demand for purine nucleotides in the growth-inhibited cultures, it can be argued that these conditions apply equally well to the *E. coli* R7AC cultures where the opposite effect on IMP biosynthesis gene expression was observed during growth in the same medium (Supplementary Fig. 2). The contrasting effects in the two species are intriguing but the underlying mechanism is yet to be determined.

Some of the genes for which expression was significantly altered during antibiotic exposure may be required to support the resistance phenotype and could be used as targets to develop ‘helper’ drugs that potentiate CTX activity^30^. The only example of antibiotic helper drugs in clinical practices are β-lactamase inhibitors. While these agents potentiate β-lactams only against resistant strains, the helper drugs conceptualized in our research would also potentiate antibiotic activity against susceptible strains, contributing to reduce the risk of treatment failure due to extrinsic non-bacterial factors. Given the strain-specific transcriptomic responses observed in our study, development of universal helper drugs that potentiate CTX against both MRSA and ESBL-producing *E. coli* may be challenging. Various histidine kinases and their associated sensor proteins were upregulated following exposure of the three strains to CTX (e.g. the histidine kinase VraS (WG79_11075) in MRSA, *atoS* and *uhpB* in *E. coli*). These sensory systems are attractive novel antibacterial targets due to their universal involvement in the regulation of bacterial stress responses^31^ and for the same reason can be regarded as promising antimicrobial helper drug targets. Putative histidine kinase inhibitors targeting the autophosphorylation domain of the protein have shown antibacterial effect across different bacterial species^32^.

Some of the processes influenced by CTX exposure (e.g. up-regulation of certain amino acid biosynthesis pathways or switching on of the cell wall stress stimulon in MRSA, and upregulation of sugar transport and metabolism in *E. coli* UR40) could be targeted to potentiate CTX activity against one of the two bacterial species. Among possible species-specific helper drug targets revealed by this study, VraX and PrsA are possible targets for CTX potentiation against MRSA since these proteins were significantly overexpressed (9.83 and 4.3 logFC, respectively) upon exposure of the MRSA strain to CTX. Overexpression of VraX was previously reported in *S. aureus* challenged by another antibiotic that targets the cell wall, vancomycin^18^. A recent study by Yan *et al*. supports the hypothesis that VraX overexpression may be a protective response for *S. aureus* survival and the protein may be involved in pathogenesis by inhibiting the classical pathway of the complement system^33^. PrsA is a peptidylprolyl isomerase that presumably plays a role in the post-translational maturation of PBP2a^34^ and is involved in the maturation of the *S. aureus* secreted virulence factor nuclease (Nuc)^35^. Therefore, both VraX and PrsA are promising targets to modulate both antimicrobial resistance and pathogenesis in this bacterial species. Cytochrome d similarly represents a potentially attractive target. A significant up-regulation in transcription of genes encoding subunits of this cytochrome in response to CTX (Fig. 3), concomitant with a marked down-regulation in expression of genes encoding the principle cytochrome c, suggest an important role for the regulation of cytochrome composition in adapting to the presence of the antibiotic. The cytochrome d family is unique to prokaryotes where it performs respiratory functions under microaerobic conditions and has previously been suggested as a general drug target for the treatment of bacterial pathogens^36^. Amongst the possible CTX helper drug targets in *E. coli*, GuaB was highly overexpressed in R7AC (4.83 log FC), and could be a candidate to potentiate CTX against β-lactamase-producing strains that display moderate levels of resistance such as this strain (MIC = 16 µg/ml). This inosine-5′-monophosphate dehydrogenase (IMPDH) plays an important role in the regulation of cell growth by catalyzing the conversion of inosine 5′-phosphate to xanthosine 5′-phosphate, the first step of guanosine 5′-monophosphate biosynthesis^37^. Even though IMPDHs are non-essential enzymes in bacteria, their inactivation affects growth and virulence in several bacterial species^38^ and diphenyl urea-based IMPDH inhibitors have been shown to have potent anti-mycobacterial activity^39^.

This study describes and explores the immediate transcriptional changes occurring in response to CTX in a diverse group of CTX-resistant pathogens, and provides a valuable set of data to help guide the search for weaknesses in bacterial defences against antimicrobial therapies. Future research is warranted to investigate whether neutralization of the presumptive helper drug target proteins identified by this study, for example by deletion of the target-encoding gene, results in significant reductions of the MIC of CTX. Specific screening assays could be designed to identify compounds interfering with the most promising targets.

## Materials and Methods

### Bacterial strains

The following CTX resistant strains previously isolated from environments relevant to public health were selected for use in the study: the ESBL-producing *E. coli* strains UR40 (ST131)^15^ and R7AC (ST297)^16^ and the methicillin-resistant *S. aureus* 55488 (ST398)^17^.

### Genome sequencing

For the two *E. coli* strains, genomic DNA was extracted for sequencing using the MasterPure™ DNA Purification Kit (Epicentre) according to the manufacturer’s instructions (with the exception of adding 3 μl (instead of 1 μl) of Ready-Lyse Lysozyme to each resuspended pellet (from 1.0 ml culture) of bacteria). *De novo* whole genome sequencing libraries were then created using the Nextera XT DNA Library Preparation Kit (Illumina), and the libraries sequenced using a 250-bp paired-end reads module on the Illumina MiSeq platform. MRSA 55488 was previously sequenced by Price *et al*.^17^, also using an Illumina platform (101-bp paired-end sequencing, Genome Analyzer IIx (GAIIx)). The average depth of sequencing coverage was 38 for strain UR40, 67 for R7AC, and 73 for 55488. The genomic assembly algorithm SPAdes was used to assemble the reference genomes from the sequencing reads, yielding 107 and 167 contigs for strains UR40 and R7AC, respectively. This compares to 45 contigs previously obtained for 55488^17^. For *E. coli* R7AC, assembled contigs that aligned to plasmid pR7AC using MUMmer were replaced with the complete plasmid sequence deposited at the National Center for Biotechnology Information (NCBI) under accession number KF434766.1. Each reference genome was annotated using the Prokaryotic Genomes Automatic Annotation Pipeline (PGAAP) developed by NCBI. ResFinder V2.1^40^ was used to identify acquired antimicrobial resistance genes. The identification was extended by a systematic manual search using the following keywords: *mecA*, methicillin, cefotaxime, cephalosporin, lactam, *bla*, *amp*, metallo and penicillin to identify genes putatively involved in CTX resistance.

### Growth and CTX treatment of bacterial cultures

The experiment was designed to assess the effects of therapeutic concentrations of CTX on mid-exponential phase cultures of the three model strains. It was estimated that a serum concentration of 30 µg/ml is achieved after 1 h following parenteral administration of 2 g of CTX to a 70 Kg person. This concentration was determined by using the pharmacokinetic-pharmacodynamic (PK-PD) mono-exponential equation known as the compartment model: *C* = *C*_0_ *∙ e*^−*k ∙ t*^ ^41,42^, where *C* is the calculated therapeutic concentration, *C*_0_ is the initial plasma concentration, *e* is the exponential factor, *k* is the elimination rate constant and *t* is the time at which the therapeutic concentration is found in serum. C_0_ was calculated from the dose distribution (Dose) divided by the volume of distribution (V_D_): C_0_ = Dose/V_D_.

Triplicate cultures of each strain in cation-adjusted Müller-Hinton broth CAMHB (Sigma-Aldrich) were incubated at 37 °C under shaking (180 rpm) until reaching mid-exponential phase (OD_600_ = 0.1, 10^7^ CFU/ml), approximately after 2 h and 40 min for *E. coli* and after 3.5 h for *S. aureus*. At this stage, each culture replicate was divided into two 3 L flasks containing 250 ml. One was treated with 30 µg/ml of CTX, while the second was used as a control. All cultures were further incubated as described above, and sampled every hour for optical density measurements and cell counts. Samples for RNA extraction were taken immediately before treatment, and 30 and 90 min after.

### RNA extraction and sequencing

Culture samples were harvested directly into double the volume of RNAprotect Bacteria Reagent (QIAGEN), incubated for 5 minutes, and then centrifuged. Cell pellets were stored at −20 °C before RNA extraction on the following day. Total RNA was isolated using RNeasy Mini columns (QIAGEN) with a modification to the manufacturer’s instructions. Briefly, cells were lysed in the presence of 300 mg acid-washed glass beads (150–600 μm diameter, Sigma) using a Fast Prep Lyser (Eppendorf) with three cycles of 1 min at maximum speed and one cycle of 7 min, cooling the samples on ice for 2 min before and between cycles. On-column DNA digestion was performed using the RNase-Free DNase Set kit (QIAGEN). RNA sample integrity was verified using a NanoDrop^TM^, (absorbance ratios 260/280 nm and 260/230 nm ≥1.8) and a Bioanalyzer 2100 (RNA Integrity Number (RIN) ≥7). Samples of total RNA (≥5 μg, measured on a Qubit) were submitted to a commercial provider (Beijing Genomics Institute) for transcriptome sequencing. Prior to sequencing, all samples were treated with DNaseI (New England Biolabs® Inc) and then enriched for mRNA molecules via ribosome-depletion using the Ribo-Zero™ magnetic Kit for bacteria (Epicentre). Sequencing was performed using an Illumina HiSeq 2000 with TruSeq V3 sequencing kits.

### Transcriptome analysis

Sequencing reads (>10 million paired-end 90 bp reads per sample) were mapped to the genome sequences using tophat v2.0.14^43^ employing the default settings but with splice awareness turned off. Mapped reads were processed and analysed in R^44^. The transcriptome analyses are based on the annotations available for these genomes on 05 July 2015. Reads mapping to annotated genome features were counted using Rsubread^45^, disallowing duplicate reads and reads that map to more than one location. Reads overlapping adjacent genes were counted for each gene. Transcripts exhibiting generally low counts were filtered out from the downstream analysis, retaining only those with >0.5 counts per million (cpm) in at least 3 of the samples. Transcript counts were TMM normalized and tested for differential expression using the limma voom method^46,47^ with limma treat^48^. PCA was performed using pcaMethods^49^, and gene ontology (GO) analysis was performed using GoSeq^50^. To identify sets of differentially expressed transcripts between CTX-exposed and control samples within each strain, the null hypothesis that the change in transcript abundance relative to the time at CTX addition (0 min) was equal in each control and CTX-exposed pair of samples was assumed according to Equation (1):

1 $$\begin{array}{l}[(\mathrm{CTX}\,\mathrm{exposed}\,{\rm{T}}30-{\rm{T}}0)-({\rm{Control}}\,{\rm{T}}30-{\rm{T}}0)=0]\\ \,{\rm{and}}\,\,[({\rm{CTX}}\,{\rm{exposed}}\,{\rm{T}}90-{\rm{T}}0)-({\rm{Control}}\,{\rm{T}}90-{\rm{T}}0)=0]\end{array}$$

A limma treat transcript fold-change threshold of 1.5 was used. For each strain, the significant (p ≤ 0.05) transcripts identified from the T30 and T90 comparisons were combined to produce a universal list of transcripts significantly changing in abundance in the 90 min period following CTX addition. The abundance profiles of significant transcripts were hierarchically clustered (Pearson correlation, using complete linkage), and the clusters subjected to GO analysis. For the GO analysis, the ontology annotations available for *E. coli* MG1655 and *S. aureus* NCTC8325 (as available from the European Bioinformatic Institute on 14 November 2016) were mapped onto the orthologues present in the strains used in this study. Orthologue identification was performed at the protein level by reciprocal best hit BLAST analysis, requiring >90% amino acid identity over >50% of the length of the protein.

### Nucleotide sequence accession numbers

The reference genome sequences are available at NCBI under the accession numbers: LAWT00000000, LAZA00000000 and LAWV00000000. The RNA-sequencing reads are available in the Short Read Archive (SRA) at NCBI under the accession numbers: SRX3171181 to SRX3171225.

## Electronic supplementary material

Supplementary Figure 1–2

Supplementary File 1

Supplementary File 2

Supplementary File 3

Supplementary File 4

Supplementary File 5

Supplementary File 6

Supplementary File 7

Supplementary File 8
