# Supplementary material for: Transcriptome analysis of extended-spectrum β-lactamase-producing Escherichia coli and methicillin-resistant Staphylococcus aureus exposed to cefotaxime
Source: Sci Rep. 2018 Oct 30;8:16076. doi: 10.1038/s41598-018-34191-3 (PMC6207760; doi:10.1038/s41598-018-34191-3)
Supplement: Supplementary file 1 — Supplementary Figure 1–2 [file 41598_2018_34191_MOESM1_ESM.pdf]

Supplementary information: Transcriptome analysis of extended spectrum β-lactamase-producing *Escherichia coli* and methicillin-resistant *Staphylococcus aureus* exposed to cefotaxime

Brochmann P. R.[a,1], Hesketh A.[b,c,1,]*, Jana B.[a], Brodersen G. H.[a] and Guardabassi, L[a,]*.

[a]University of Copenhagen, Faculty of Health and Medical Sciences, Department of Veterinary and Animal Sciences, Frederiksberg, Denmark

[b]University of Cambridge, Department of Biochemistry and Cambridge Systems Biology Centre, Cambridge, United Kingdom

[c]University of Brighton, School of Pharmacy and Biomolecular Sciences, Brighton, United Kingdom

Running title: Transcriptome of resistant bacteria exposed to cefotaxime

[1]Brochmann P. R. and Hesketh A. equally contributed to the work.

*Address correspondence to Andrew Hesketh (a.hesketh@brighton.ac.uk) and Luca Guardabassi (lg@sund.ku.dk).

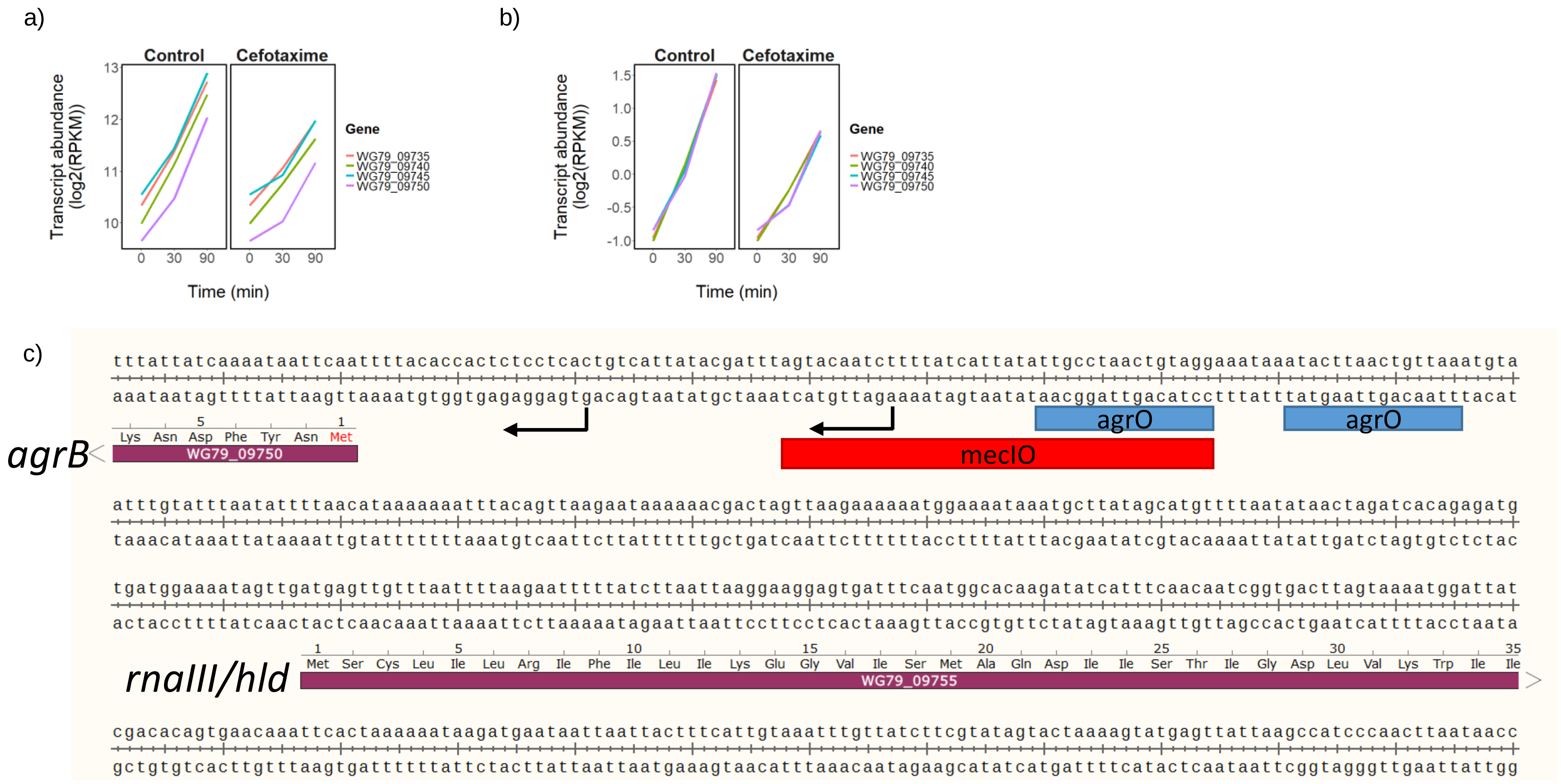

Supplementary Figure 1. Transcription of the genes assigned to the accessory gene regulator (*agr*) operon in *S. aureus* 55488 (*agrA*, WG79_09735; *agrC*, WG79_09740; *agrD*, WG79_09745; *agrB*, WG79_09750. a) Normalized log2(RPKM) values; b) Mean centred values from a); c) Promoter sequence of *agrB* (the first gene in the *agr* operon) showing the location of the same operator sequences (colored boxes) and transcriptional start sites (arrows) as were identified in MRSA strain USA300 by Choe et al. 2018.

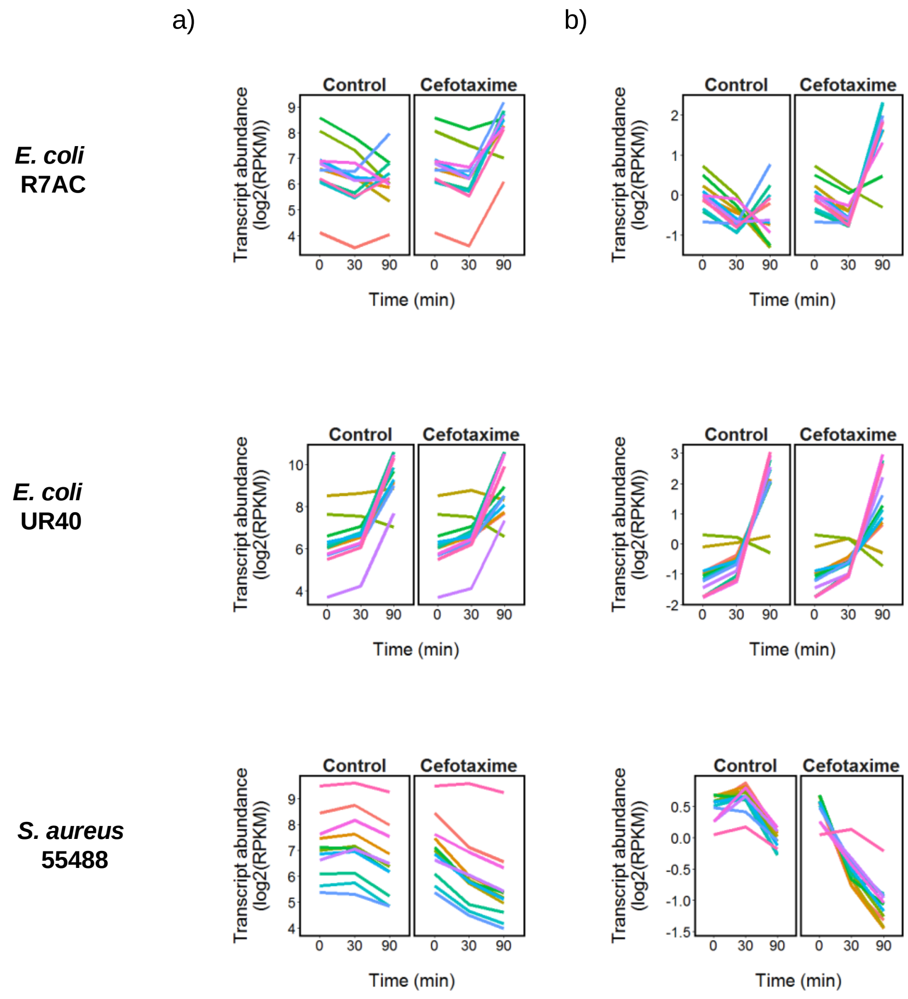

Supplementary Figure 2. Transcription of the genes assigned to the de novo IMP biosynthesis GO category (GO: 0006189) in the three strains. a) Normalized log2(RPKM) values; b) Mean centered values from a).
